# Concomitant Uterine and Bilateral Adnexal Torsion in a Postmenopausal Woman: A Case Report

**DOI:** 10.7759/cureus.74428

**Published:** 2024-11-25

**Authors:** Annette Van Swaay, Jorge Garibay

**Affiliations:** 1 Obstetrics and Gynecology, Saint Joseph Hospital, Denver, USA; 2 Obstetrics and Gynecology, University of Colorado School of Medicine, Denver, USA

**Keywords:** adnexal torsion, case report, pelvic pain, postmenopausal, uterine torsion

## Abstract

Adnexal torsion is a well-recognized gynecologic emergency; however, uterine torsion is less well-known. The majority of uterine torsions occur in gravid uteri; torsion in postmenopausal patients is rare. We report a case of uterine and bilateral adnexal torsion in a postmenopausal woman due to a large leiomyoma. This patient presented to the emergency department with acute onset pelvic pain in the setting of a known pelvic mass. Imaging findings were significant for a large pelvic mass that differed in location when compared to prior imaging. In addition, the cervix had a "twisted" appearance on imaging. Overall these findings were suspicious for uterine torsion, and surgical management was planned. Operative findings were significant for 720-degree uterine torsion at the level of the lower uterine segment due to a large subserosal fibroid extending into the left broad ligament. Pathology was significant for confirmed leiomyoma measuring 25 cm, with edema and myxoid changes, uterus and bilateral ovaries with hemorrhage and congestion, all of which supported the diagnosis of uterine torsion, which also resulted in bilateral adnexal torsion. This case demonstrates an instance of uterine and bilateral adnexal torsion that was managed in a timely fashion. If surgical treatment is delayed, grave sequelae can occur. Consideration of concomitant uterine and adnexal torsion during an acute pelvic pain workup is crucial to ensure appropriate inpatient management, preoperative counseling, surgical consent, and patient safety.

## Introduction

Adnexal torsion is a well-recognized gynecologic emergency; however, uterine torsion is less well-known. Uterine torsion is defined as a rotation of >45 degrees around the long axis of the uterus, usually at the level of the isthmus. Most case reports of uterine torsion have occurred in gravid uteri, and torsion of uteri in non-pregnant patients is rare but reported [1]. Uterine torsion in postmenopausal patients is usually related to underlying pelvic pathology. It is important to also consider borderline and malignant ovarian masses, in addition to benign pathology, in the differential diagnosis of a pelvic mass in a postmenopausal woman. The majority of borderline tumors (68%) are found in postmenopausal women; they can vary greatly in size from 3 to 40 cm. Mucinous tumors are the most common subtype (68%), followed by serous (24%) and other (4%) [2]. Consideration of malignancy should include evaluating tumor markers and looking for hallmark signs of malignancy on imaging. Timely consideration and diagnosis of uterine torsion during an acute pelvic pain workup is important to ensure prompt treatment and avoidance of dangerous sequelae.

## Case presentation

A 67-year-old postmenopausal woman presented to the hospital for acute pelvic pain with a known pelvic mass. The patient provided consent for the publication of this case report. The pelvic mass of unclear origin had been previously found on computed tomography (CT) imaging when the patient presented for abdominal pain at another facility. She had previously been evaluated as an outpatient one year prior and had normal tumor markers, including CEA, Ca 19-9, and Ca-125, due to concern for an adnexal mass. At that time, she was scheduled but unable to receive surgery due to hypertensive urgency. The patient was then lost to follow-up.

**Figure 1:**
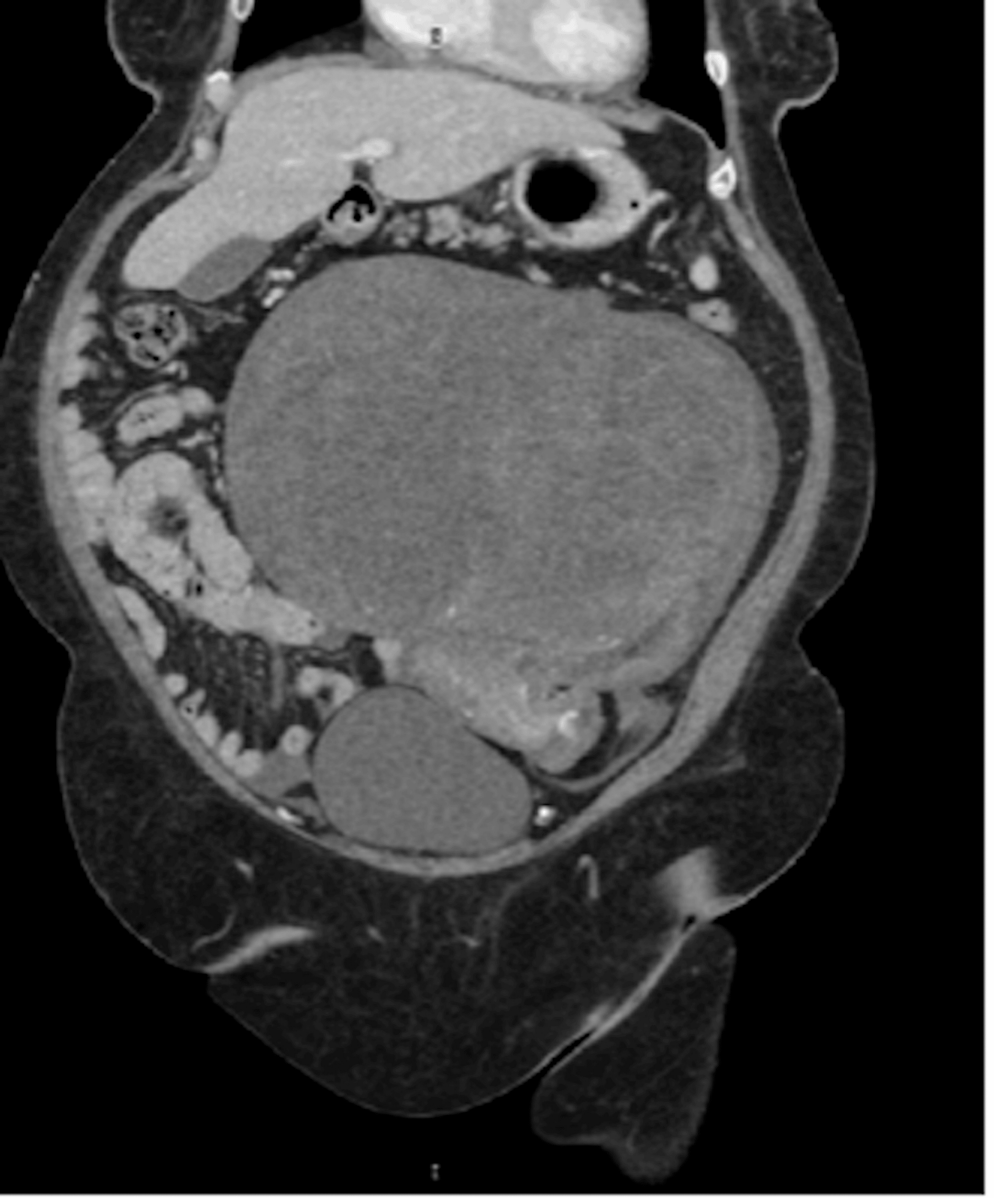
Large pelvic mass on CT imaging

In the emergency department, the patient reported that pelvic pain was of acute onset and had been worsening. The patient also endorsed multiple episodes of emesis. A physical exam revealed a distended abdomen with a large, firm mass extending into the upper abdomen, approximately 25 cm in size and angling into the left lower quadrant. Laboratory studies were notable for a lactate that peaked at 3.3 mmol/L. A CT of the abdomen/pelvis was performed, which showed a large mass arising from the left pelvis that was proved to represent a large uterine fibroid that measured 25.2 x 13.6 x 17.5 cm, not a significant change from the CT scan performed a year ago (Figure 1).

**Figure 2:**
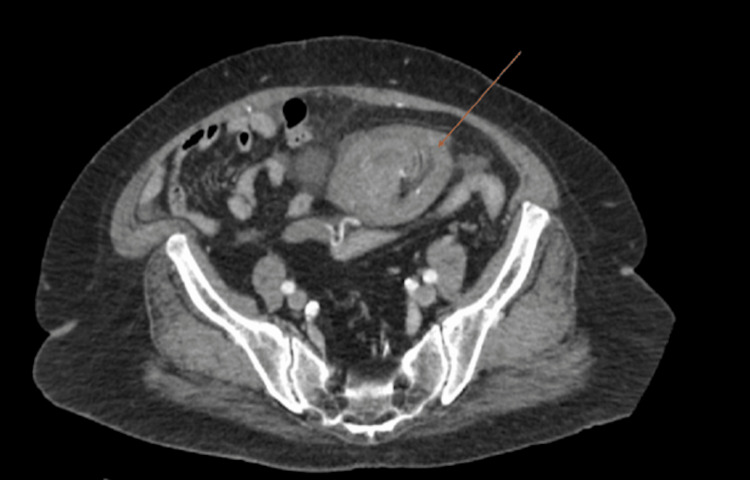
CT scan demonstrating whorled and twisted appearance of the cervix (indicated by an arrow)

The lower uterine body/cervix appeared twisted/swirled (Figure 2), and the suspected fibroid was displaced more medially in the abdomen, which was located in the left abdomen on prior imaging. The ovaries were not clearly identified. The correlation of these findings was concerning for uterine torsion. Medical history was significant for bipolar disorder, hypertension, type 2 diabetes, chronic obstructive pulmonary disease/asthma, a history of supraventricular tachycardia, and a body mass index of 33. Surgical history was significant for one cesarean section via vertical incision. The patient was admitted and medically optimized for surgery due to uncontrolled hypertension and diabetes. The pain was controlled with scheduled Tylenol and morphine as needed. The patient consented to an exploratory laparotomy and total abdominal hysterectomy with bilateral salpingo-oophorectomy due to the suspected uterine or adnexal torsion.

**Figure 3:**
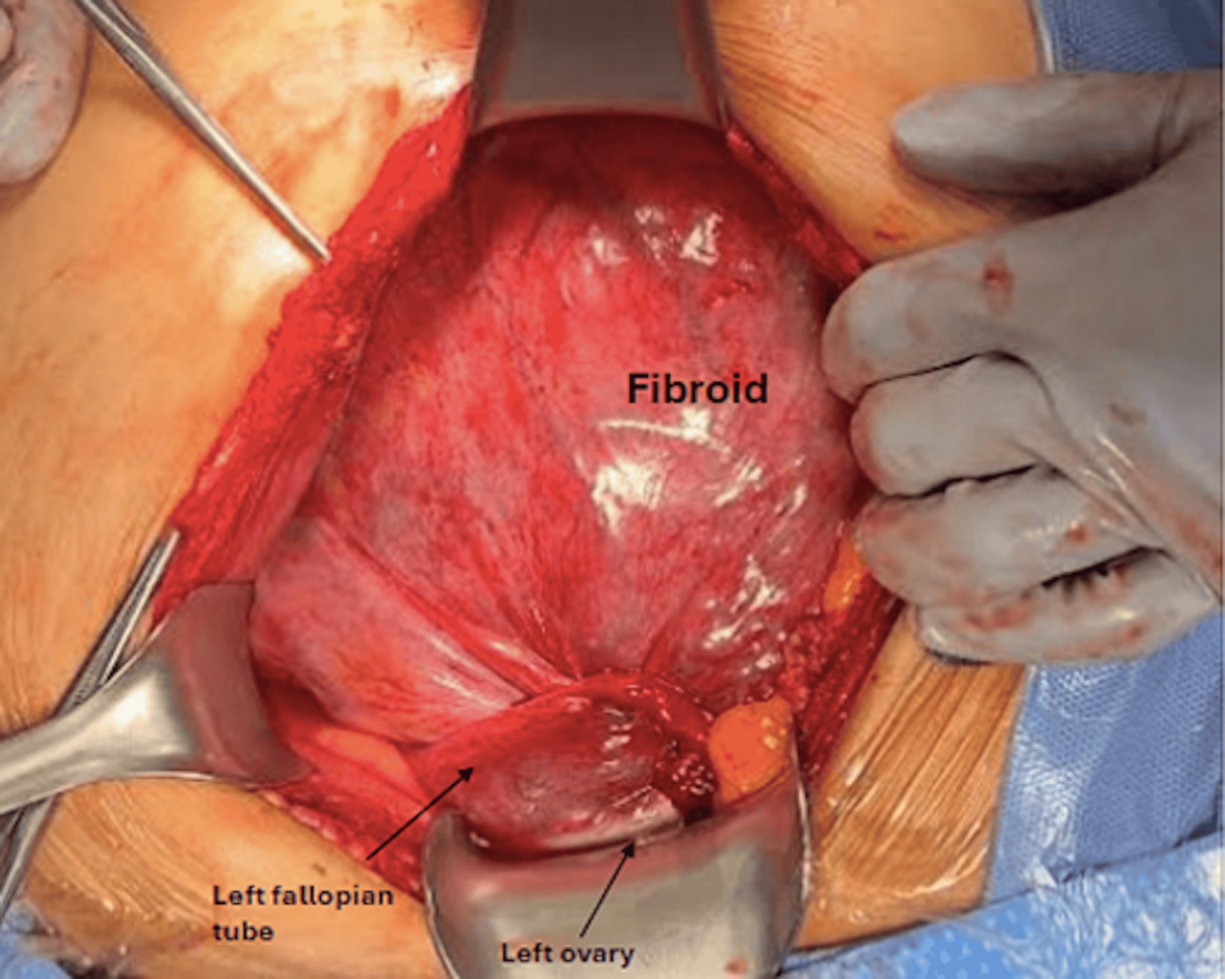
Operative findings of complete uterine and bilateral adnexal torsion

On hospital day 2, the patient underwent an exploratory laparotomy, pelvic washing, total abdominal hysterectomy with bilateral salpingo-oophorectomy, and cystoscopy. Due to the size of the mass, this was done via a vertical midline incision. Operative findings were significant for 720-degree torsion due to what appeared to be a large subserosal fibroid extending into the left broad ligament and adherent to the left ovary (Figure 3).

**Figure 4:**
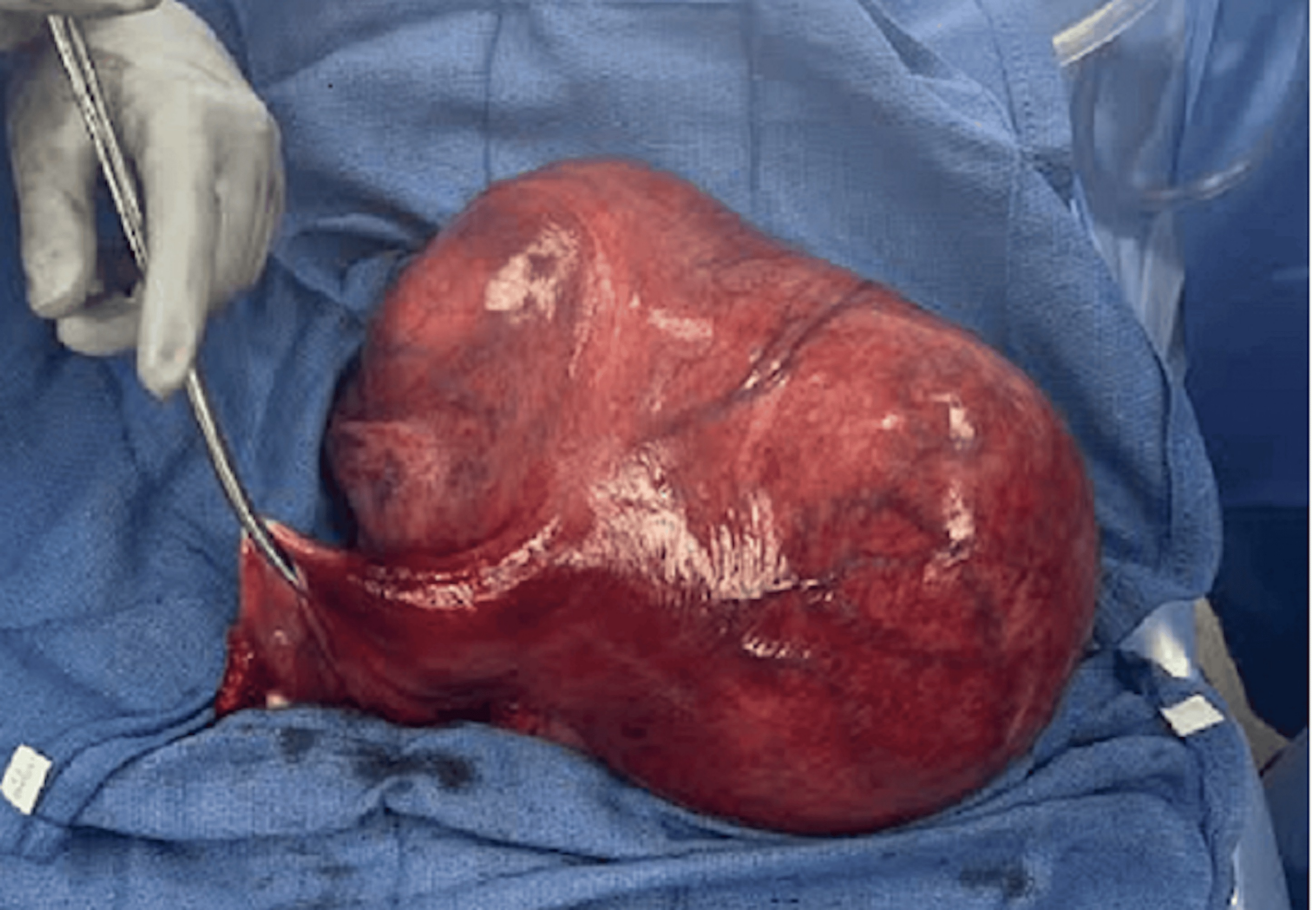
Specimen after de-torsion

A uterus could not be appreciated separate from the fibroid. At the time of surgery, the left adnexa appeared to be involved in the torsion, while the right adnexa appeared grossly normal. De-torsion was performed upon entry (Figure 4), and the surgery was uncomplicated. The total specimen weight was 3738 g. Final pathology was significant for confirmed leiomyoma measuring 25 cm, uterus with atrophic endometrium, hemorrhage, and congestion, and bilateral ovaries with hemorrhage and congestion which supported the diagnosis of uterine torsion, also resulting in bilateral adnexal torsion. The patient had an unremarkable postoperative course and was discharged on hospital day 5.

## Discussion

This case demonstrates the rare occurrence and challenging diagnosis of a concomitant uterine and bilateral adnexal torsion in a postmenopausal woman.

Uterine torsion is an uncommon event. Case reports describe the majority of uterine torsions occurring during pregnancy; however, it has also been observed in adolescent and postmenopausal patients [1]. In postmenopausal patients, only seven cases were reported between 2010 and 2020 [3]. A fibroid uterus is the most common abnormality seen in non-gravid uterine torsion. In our patient, surgical and pathological findings confirmed the insulting pathology to be a large subserosal fibroid extending into the left broad ligament and adherent to the left ovary. However, preoperatively the origin of the pelvic mass could not be definitively discerned. In a postmenopausal patient, it is important to consider borderline and malignant ovarian masses in a differential diagnosis. Borderline tumors are more common in postmenopausal women and present unilaterally 66% of the time. The size of our patient's mass could have also been consistent with a borderline tumor, as they can vary in size from 3 cm to 40 cm [2]. A malignant ovarian mass was reasonably ruled out in our patient due to the lack of interval change in serial imaging, normal tumor markers, and lack of red flag findings on imaging. Another proposed risk factor is the history of cesarean section. It is thought that poor healing of the isthmus after cesarean delivery may predispose to uterine torsion, especially in the setting of uterine pathology, which our patient had both [4]. In our case, the location and size of the fibroid predisposed this patient to have uterine torsion as well as adnexal torsion.

Symptomology of uterine torsion can vary widely, ranging from acute to chronic to asymptomatic. The most common presenting symptom is abdominal pain, which can vary significantly from person to person. As the uterus becomes engorged, the size can increase and cause a mass effect on nearby organs, leading to abdominal pain, nausea, vomiting, constipation, and diarrhea. If torsion persists for a long period of time, the uterus can become gangrenous, leading to sepsis [5]. We believe our patient’s symptoms were exacerbated due to the combination of uterine and adnexal torsion, which expedited the decision for surgical management.

Ultrasound has long been established as the imaging modality of choice for gynecologic evaluation; however, it can be limited in the evaluation of uterine torsion. It can be useful if there is a prior ultrasound for comparison, in which the position of the fibroids could be noted to have changed. In our case, the radiologist noted a change in the position of the large mass when compared to prior CT imaging. When comparing imaging studies, a CT scan is likely the most useful imaging modality for the evaluation of uterine torsion. Multiple case reports, including our patient, have CT scans demonstrating a “whorled structure” representing the twisted cervix and a lack of contrast enhancement within the uterus representing infarction [1]. Magnetic resonance imaging can show a change in the upper vagina from the normal H shape to an X shape, suggesting twisting of the tissue [6]. Due to the grave consequences that can occur with untreated uterine torsion, as well as the wide variety of clinical presentations, it is important to always consider uterine torsion in the differential diagnosis of pelvic pain. Uterine torsion should be even more carefully considered in the setting of large leiomyoma. We recommend performing CT imaging in cases of pelvic pain with unclear etiology to evaluate for findings consistent with uterine torsion.

A definitive diagnosis of uterine torsion can only be made at the time of surgery, and the definitive treatment of non-gravid uterine torsion is a hysterectomy. If management is delayed, grave sequelae including shock, coagulopathy, and sepsis can occur. In perimenopausal or postmenopausal patients, hysterectomy is the preferred treatment. In younger patients who desire childbearing, conservative surgery can be considered depending on the viability of the uterus. Conservative surgery would involve myomectomy or cystectomy to address the insulting pathology, as well as consideration of plication or round or uterosacral ligaments to minimize the risk of recurrent uterine torsion [6].

## Conclusions

This case report demonstrates a confirmed case of uterine and bilateral adnexal torsion in a postmenopausal patient that was highly suspected based on imaging. Interval change of CT imaging was key to establishing the diagnosis of uterine torsion. If untreated, uterine torsion can lead to shock, coagulopathy, or sepsis. These potential grave sequelae and the surgical complications that can result from operation on a gangrenous uterus and adnexa highlight the need for timely diagnosis and intervention. It is important to always consider uterine torsion and possible concomitant adnexal torsion in the differential of a pelvic pain workup, especially in the setting of a large pelvic mass. The utilization of CT imaging to aid in diagnosis should also be considered, as this is more helpful than traditional gynecologic ultrasound imaging. Further research in laboratory abnormalities and CT and MRI techniques may enhance the diagnosis of uterine torsion in the future.
